# A Case of Spinal Epidural Extramedullary Hematopoiesis Diagnosed Using Indium-111 Bone Marrow Scintigraphy

**DOI:** 10.7759/cureus.84940

**Published:** 2025-05-28

**Authors:** Haruka Tanaka, Masashi Nakadate, Hiroyuki Inose, Kazunori Kubota

**Affiliations:** 1 Department of Radiology, Dokkyo Medical University Saitama Medical Center, Saitama, JPN; 2 Department of Orthopaedics, Dokkyo Medical University Saitama Medical Center, Saitama, JPN

**Keywords:** extramedullary hematopoiesis, in-111 bone marrow scintigraphy, nuclear medicine, radiotherapy, spinal epidural tumor

## Abstract

Although indium-111 (^111^In) bone marrow scintigraphy is commonly used to assess bone marrow function, it plays a significant role in diagnosing extramedullary hematopoiesis (EMH) due to isotope accumulation in hematopoietic bone marrow. In our case, a 67-year-old man with polycythemia vera presented with an epidural tumor, for which EMH was considered a differential diagnosis. The diagnosis was subsequently confirmed by ^111^In bone marrow scintigraphy. The tumors exhibited a reduction in size following radiation therapy. We report this case to highlight the utility of ^111^In bone marrow scintigraphy in diagnosing EMH. This imaging modality can confirm epidural EMH and help avoid unnecessary surgeries.

## Introduction

Although indium-111 (^111^In) bone marrow scintigraphy is primarily used to evaluate bone marrow function, it is also useful for diagnosing extramedullary hematopoiesis (EMH) because the isotope accumulates in the hematopoietic bone marrow [1]. EMH typically occurs in the liver or spleen but can also arise in various other locations [2]. Previous reports have documented the use of bone marrow scintigraphy to diagnose EMH in the intrathoracic space [3], paravertebral region [4], presacral area [5], intracranial space [6], and lungs [7]. However, there have been no reports on its use in spinal canal imaging.

We report a case of spinal epidural EMH diagnosed using ^111^In bone marrow scintigraphy, highlighting its potential as a noninvasive diagnostic tool that may guide appropriate treatment and help avoid unnecessary surgery.

## Case presentation

The patient was a 67-year-old man with a history of polycythemia vera, hypertension, and gout. He was referred to our hospital after experiencing back pain for six months. 

Initial laboratory tests revealed elevated white and red blood cell counts (Table 1). Neurological symptoms were limited to increased bilateral patellar tendon reflexes. No muscle weakness was observed in the lower limbs during the initial visit.

**Table 1:** Initial laboratory test results with reference ranges

Laboratory test	Result	Units	Reference range
White blood cell (WBC)	10100	/µL	3300–8600
Red blood cell (RBC)	7.96	million/µL	4.35-5.55
Platelet	159000	/µL	15800-348000
Hemoglobin	19.4	g/dL	13.7-16.8
Hematocrit	63.6	%	40.7-50.1
Mean corpuscular volume (MCV)	79.9	fL	83.6–98.2
Lactate dehydrogenase (LDH)	260	U/L	124–222
C-reactive protein (CRP)	0.24	mg/dL	<0.14

**Figure 1:**
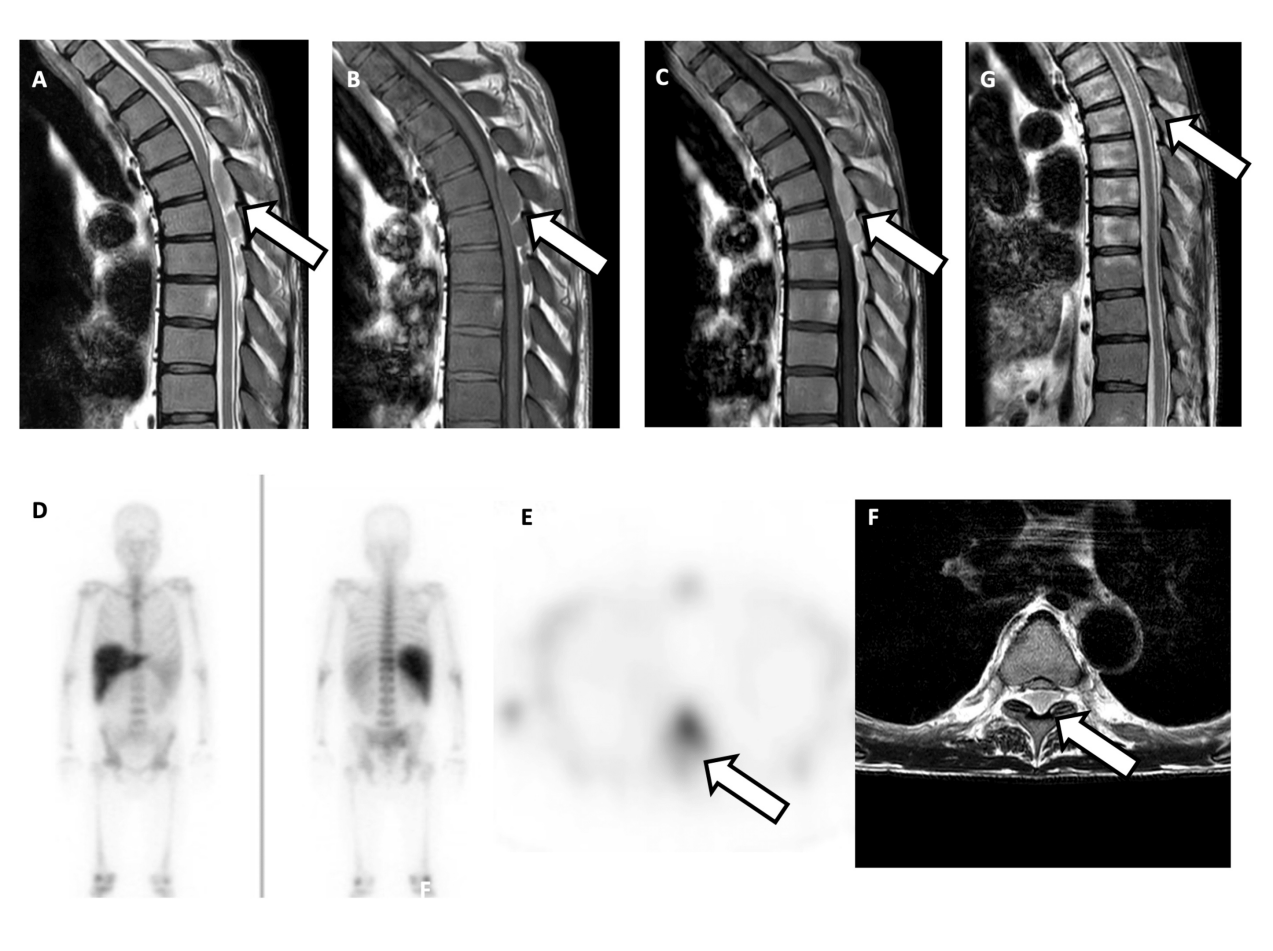
A-G:MRI and In-111 bone marrow scintigraphy A-C: The posterior epidural mass within the spinal canal compresses the dural sac and causes spinal cord compression, as indicated by the arrows. D-F: Although not visualized on planar bone marrow scintigraphy, faint radiotracer accumulation corresponding to the mass is observed on SPECT imaging, marked by arrows. G: The epidural mass decreased in size following radiation therapy, as shown by the arrow. A: T2WI sagittal view, B: T1WI sagittal view, C: Gd-T1WI sagittal view, D: ^111^In bone marrow scintigraphy planar imaging, E: ^111^In bone marrow scintigraphy SPECT imaging, F: T2WI axial view, G: Post-treatment T2WI sagittal view. Abbreviations: Gd-T1WI, gadolinium-enhanced T1-weighted image; T1WI, T1-weighted image; T2WI, T2-weighted image; SPECT, single-photon emission computed tomography

Magnetic resonance imaging (MRI) revealed extradural masses at the Th5-7 and L3-4 levels, showing low signal intensity on T1-weighted imaging (T1WI) and slightly higher signal intensity than that of the spinal cord on T2-weighted imaging (T2WI), accompanied by contrast enhancement (Figure 1A-1C). High signal intensity was observed on diffusion-weighted imaging, along with a slight decrease in the apparent diffusion coefficient. The dural sac was compressed by the mass. The spine demonstrated diffuse low signal intensity on T1WI, suggesting hyperhematopoietic activity and fibrosis.

Given the patient’s medical history and lack of signal change over a four-month period, EMH was suspected, and ^111^In bone marrow scintigraphy was performed. In planar imaging, tracer accumulation was not prominently visualized (Figure1D). However, single-photon emission computed tomography (SPECT) revealed slight accumulation corresponding to the mass (Figure1 E-F), and EMH was considered highly likely. Approximately two months after the initial visit, the patient began to experience progressive paralysis in both lower limbs, and radiation therapy was administered to alleviate the symptoms. Radiotherapy was administered at a total dose of 20 Gy in 10 fractions of 2.0 Gy.

MRI performed one month after radiation therapy showed a reduction in the mass (Figure1 G).

The tumor shrank, and symptoms such as lower limb muscle weakness and back pain were alleviated. MRI performed six months post-treatment demonstrated sustained reduction in the mass, with no evidence of symptom progression.

## Discussion

EMH most commonly occurs in the liver and spleen but can also develop in various other parts of the body [2-7]. However, occurrences within the spinal canal are uncommon [8], and the underlying cause remains unclear. Several hypotheses have been proposed, including hematogenous spread of hematopoietic cells from the vertebral medulla, embolic deposition of these elements into the intraspinal space, and reactivation of embryonic stem cells originating in the spinal region [8]. 

The differential diagnosis of extramedullary spinal tumors includes not only EMH but also hemangiomas, infections such as abscesses, hematomas, discogenic diseases (e.g., disc herniation, synovial cysts, and post-surgical epidural fibrosis), and metastatic tumors [9]. Differentiating EMH from other pathologies is essential and typically involves a combination of noninvasive techniques, including computed tomography (CT), MRI, and nuclear medicine imaging, as well as invasive procedures, such as biopsy. On MRI, active EMH lesions typically show intermediate signal intensity on both T1WI and T2WI, higher than that of the bone marrow, and exhibit gadolinium enhancement. By contrast, old inactive lesions may show either high or low signal intensity on both T1WI and T2WI due to fatty infiltration or iron deposition, respectively, and demonstrate reduced contrast enhancement [10]. 

Nuclear medicine imaging plays a particularly important role in diagnosing EMH, with ^111^In bone marrow or ^99m^Tc Sn colloid scintigraphy commonly performed for this purpose [11]. EMH is usually associated with hematological disorders such as myelofibrosis, myelodysplastic syndromes, thalassemia, polycythemia vera, leukemia, and lymphoma [12]. According to the Mayo Clinic database, approximately 83% of patients with EMH have an underlying myeloproliferative neoplasm [13]. In the present case, the patient had polycythemia vera, making EMH a reasonable differential diagnosis. However, a definitive diagnosis was difficult based on the initial MRI alone; therefore, ^111^In bone marrow scintigraphy was performed. Using planar imaging alone, it was not easy to determine whether the tumor had tracer uptake distinct from the normal vertebral bone marrow; however, the addition of SPECT imaging enabled differentiation between normal bone marrow and abnormal accumulation, thereby providing valuable diagnostic information. 

Spinal cord compression is a significant concern when EMH occurs within the spinal canal. Thoracic spine involvement is particularly problematic because this region has a narrow spinal canal and limited motility, increasing the risk of severe neurological deficits [14]. Currently, no established treatment protocols exist for EMH. Treatment options include surgical decompression or a combination of surgery and postoperative radiation therapy [15]. EMH is highly sensitive to radiation, and tumor shrinkage has been reported following radiation therapy, particularly with low-dose radiation regimens [15,16]. Cailleteau et al. [15] reported a case of spinal canal EMH treated with radiation therapy. They found EMH in a region similar to that in our case and observed that low-dose radiation therapy (18 Gy delivered in 10 fractions of 1.8 Gy) improved neurological symptoms. They suggested a total dose of 16-20 Gy using the classical fractionation regimen (1.8-2 Gy per fraction).

Using bone marrow scintigraphy to confirm the diagnosis of EMH may help avoid the need for invasive surgical procedures, and radiation therapy is expected to reduce tumor size.

## Conclusions

This case illustrates the importance of considering EMH as a differential diagnosis for spinal epidural masses, particularly in patients with underlying hematologic disorders. ^111^In bone marrow scintigraphy enables a non-invasive diagnosis and guides effective treatment. SPECT imaging, in particular, plays an important role in evaluating intraspinal lesions. The use of this imaging modality may reduce the need for invasive biopsy or surgery. Clinicians should include EMH in the diagnostic workup for similar cases.
